# Luminescence of mesoporous silicon powders treated by high-pressure water vapor annealing

**DOI:** 10.1186/1556-276X-7-382

**Published:** 2012-07-11

**Authors:** Bernard Gelloz, Armando Loni, Leigh Canham, Nobuyoshi Koshida

**Affiliations:** 1 Graduate School of Engineering, Nagoya University, 2-24-16 Furo-cho, Chikusa-ku, Nagoya, Aichi 464-8603, Japan; 2 Intrinsiq Materials Ltd., Malvern Hills Science Park, Geraldine Road, Malvern, Worcs, WR14 3SZ, UK; 3 Graduate School of Engineering, Tokyo University of Agriculture and Technology, 2-24-16 Nakacho, Koganei, Tokyo 184-8588, Japan

**Keywords:** Porous silicon, Luminescence, Powder

## Abstract

We have studied the photoluminescence of nanocrystalline silicon microparticle powders fabricated by fragmentation of PSi membranes. Several porosities were studied. Some powders have been subjected to further chemical etching in HF in order to reduce the size of the silicon skeleton and reach quantum sizes. High-pressure water vapor annealing was then used to enhance both the luminescence efficiency and stability. Two visible emission bands were observed. A red band characteristic of the emission of Si nanocrystals and a blue band related to localized centers in oxidized powders. The blue band included a long-lived component, with a lifetime exceeding 1 sec. Both emission bands depended strongly on the PSi initial porosity. The colors of the processed powders were tunable from brown to off-white, depending on the level of oxidation. The surface area and pore volume of some powders were also measured and discussed. The targeted applications are in cosmetics and medicine.

## Background

Nanocrystalline porous silicon (PSi) has been extensively studied for its many useful properties, in particular, in photonics [1-3]. Even though bulk silicon can only emit light in the near-infrared, PSi can emit visible light because of quantum effects taking place in its nanostructure. Band to band recombinations in bulk silicon are indirect, leading to very low luminescence efficiency. In PSi, the luminescence efficiency can be relatively high, in principle, thanks to high exciton localization in silicon nanocrystals and more direct transitions. However, the nanocrystal surface must be defect-free for light emission to be effective, and it must be stable for any application to be considered. In practice, this is not easy to achieve and has been a major challenge for PSi. Recently, efficient and stable red photoluminescence (PL, 23 % external quantum efficiency) [4,5] has been obtained from lightly-doped p-type PSi treated by high-pressure water vapor annealing (HWA). Stable electroluminescence from heavily-doped n-type PSi was also demonstrated [6]. HWA has also been shown to greatly reduce surface recombination in silicon photonic crystals [7].

More recently, efficient blue PL from partially oxidized PSi was also reported [8]. This blue emission includes an intrinsic blue phosphorescence band which exhibits a unique relatively long lifetime (several seconds) because of radiative transitions from triplets to ground states [9].

PSi is a biocompatible material [10] now under clinical evaluation as a therapeutic biomaterial [11]. Consequently, it can be used for biological and medical applications and even cosmetic treatments [12] if desirable optical and luminescent properties can be achieved at low enough cost.

Most reports about PSi focus on layers rather than powders. Nanosilicon powders are required in various fields such as drug delivery [3] and cosmetics [12]. In this paper, we report a study of the luminescence properties of PSi powders and the effect of HWA. In order to limit the production cost, relatively heavily doped Si was used (no need for back contact processing for anodization). Nevertheless, powders derived from such single crystal silicon feedstocks are still considered ‘model’ structures, rather than those that would be ultimately used in high-volume applications. HWA was used to stabilize and enhance the luminescence efficiency of the powders.

## Methods

PSi layers were fabricated on 6-in diameter p-type silicon wafers (resistivity 0.01 to 0.1 Ω·cm) by electrochemical anodization in hydrofluoric acid- methanol electrolyte. The current densities and anodization times were chosen to obtain initial porosities of 55 % and 65 %. The PSi layers were then detached from each substrate, in situ, by application of a high-current density step; complete detachment was achieved by subsequent immersion/rinsing in methanol. After drying, the membranes were then fragmented and hand-milled to form PSi powders.

In order to increase the porosity of those powders and to yield a reduced silicon skeletal size for increased PL efficiency, the as-anodized powders were chemically leached using a solution of HF and methanol.

The powders were treated by HWA [4,5] at 4.5 MPa at 260 °C for 18 h. This oxidizing step further decreases the size of the silicon skeleton and provides an effective surface passivation by relaxed and good quality thin oxide.

The PL was acquired using a fiber-optic spectrometer (Hamamatsu C10029, Hamamatsu Photonics K.K., Japan). The excitation was either the 325-nm line of a CW HeCd laser or the fourth harmonic (266 nm) of an yttrium aluminum garnet laser (12 ps pulse duration, 10 Hz repetition rate, acquisition of one spectrum included 100 pulses). Low temperature measurements were done, while the powders were in a cryostat under vacuum. Surface area and pore volume were determined with the nitrogen gas adsorption method using a Micromeritics Tristar 3000 instrument (Micromeritics Japan, G.K., Chiba, Japan).

## Results and discussion

**Figure 1:**
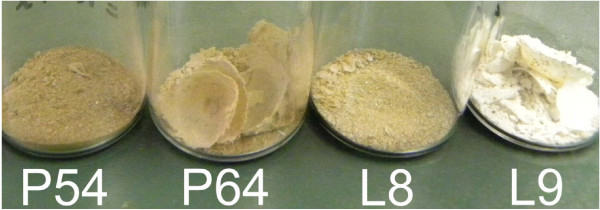
**Pictures showing different PSi powders after HWA treatment.** Powder names, as defined in Table 1, are written below each sample.

The color under room lighting or sky light is an important material property in cosmetics. Facial cosmetic products like foundation, for example, are typically tunable shades of light brown. The effect of HWA and initial PSi porosity on the color of the PSi powder can be seen in Table 1 and Figure 1. The as-formed powders are dark brown, regardless of initial porosity. After HWA, powders appear light brown, except for the sample L9 (initial porosity 84 %), which looks off-white. The color of a material depends on many factors. For PSi powders, in some part of which, quantum confinement may occur; wavelength-dependent light absorption and scattering are particularly important. HWA modifies these properties mainly by partially oxidizing PSi. The potential effects of oxidation in PSi are (i) shifting the absorption edge towards shorter wavelengths (Si oxide bandgap being much wider than that of silicon) and (ii) reduction of absorption due to enhanced scattering due to the material size increase induced by oxidation and evidenced by BET. The light-brown color was observed in most PSi powders after HWA can be attributed to these effects. The off-white color of sample L9 is an extreme case, where most visible light is scattered back to the observer. In this case, most of the PSi structure was oxidized, leading to a kind of porous silica powder, which absorbs much less than PSi and efficiently scatters visible light. HWA is a self-limited oxidation process. It produces an oxide layer of a few nanometer in thickness at the surface of PSi. However, for sample L9, the initial porosity was very high (84 %), meaning that the silicon walls within the PSi structure were very thin. Thus, most of the silicon in this powder was oxidized by HWA.

**Table 1:** HWA effect on the color of PSi powders as a function of porosity

**Name**	**Porosity**	**Initial color**	**Color after HWA**
P54	54 %	Dark brown	Light brown
P64	64 %	Dark brown	Light tan
L8	69 %	Dark brown	Light tan
L9	84 %	Dark brown	Off-white

Colors of PSi powders before and after HWA. Actual pictures of the powders can be seen in Figure 1.

BET analysis of as-formed powders shows values of the surface areas and pore volume (Table 2) of samples L8 and L9. After HWA, both quantities have significantly decreased, roughly by an order of magnitude. This can be attributed to the volume expansion of the material due to HWA-induced oxidation and some pore coalescence resulting in significantly smaller surface area and pore volume.

Surface area, pore volume, and average pore diameter of samples L8 and L9 before and after HWA. The results were obtained using the BET technique. The percentage values are the initial porosities of the samples.

**Figure 2:**
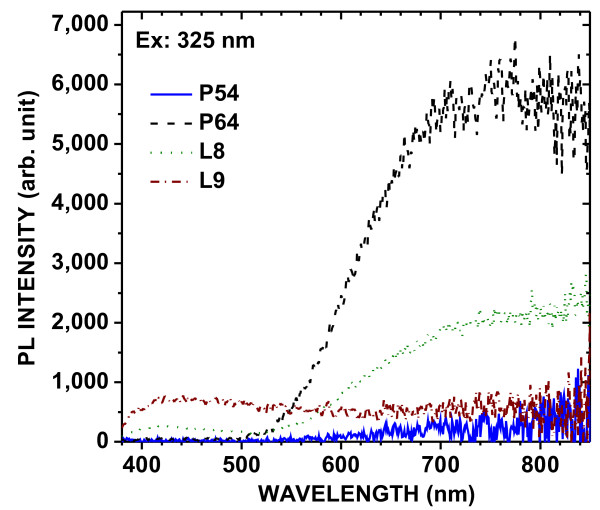
PL excited at 325 nm, at 300 K, of different PSi powders after HWA treatment.

**Figure 3:**
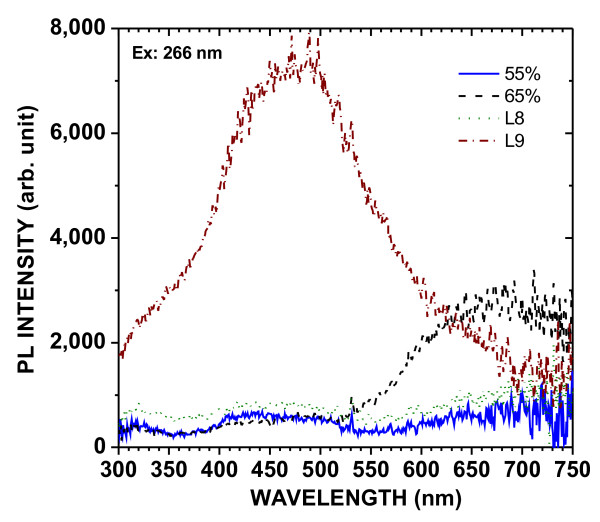
PL excited at 266 nm, at 300 K, of different PSi powders after HWA treatment.

The PL spectra of the powders under excitation at 325 and 266 nm are shown in Figures 2 and 3, respectively. Three PL bands were observed in all samples: a UV band (*λ* < 380 nm), a blue band (380 nm < *λ* < 550 nm), and a red band (*λ* > 550 nm). The red band is attributed to exciton recombination in quantum-confined nanocrystals [1]. The blue band is similar to that observed in HWA-treated PSi layers [8] and can, therefore, be attributed to localized centers associated with the presence of oxide in the nanostructures. The weak UV band is likely due to centers in the oxide. The blue band is more efficiently excited at 266 nm than at 325 nm, as observed previously for PSi layers [9]. Indeed, this excitation of this band is characterized by a large Stokes shift.

The initial porosity has a great influence on the PL. The intensity of the red band increases with the initial porosity from 54 % to 64 % and then decreases for higher porosities (69 % and 84 %). The increase in PL intensity is attributed to increased quantum confinement (size decrease) due to HWA-induced silicon oxidation. However, for high porosities (69 % and 84 %), the initial nanocrystals are already rather small, and most of them were fully oxidized by HWA, thus leading to lower intensity of the red band.

The blue band is observed in all powders when the excitation wavelength is 266 nm. Its intensity increases with the initial PSi porosity. It is strikingly high for powder L9. This powder has been very heavily oxidized by HWA, as confirmed by the off-white color of the final product, as opposed to the brown color of other powders. Therefore, this strong blue PL is clearly related to the oxide.

**Figure 4:**
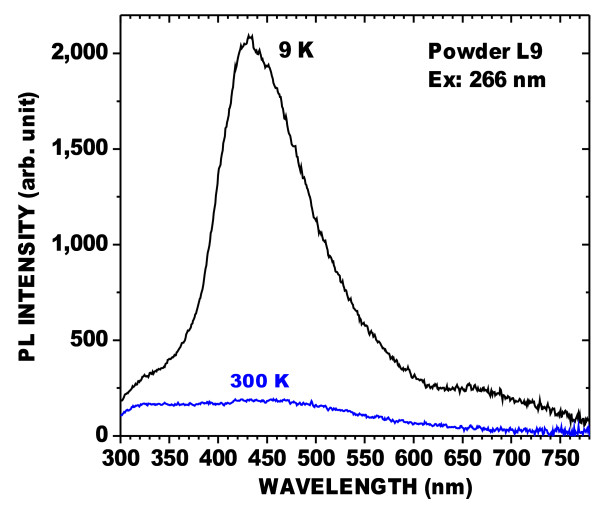
**PL spectra of powder L9 after HWA treatment.** Excited at 266 nm, taken at 9 and 300 K, as indicated in the figure.

**Figure 5:**
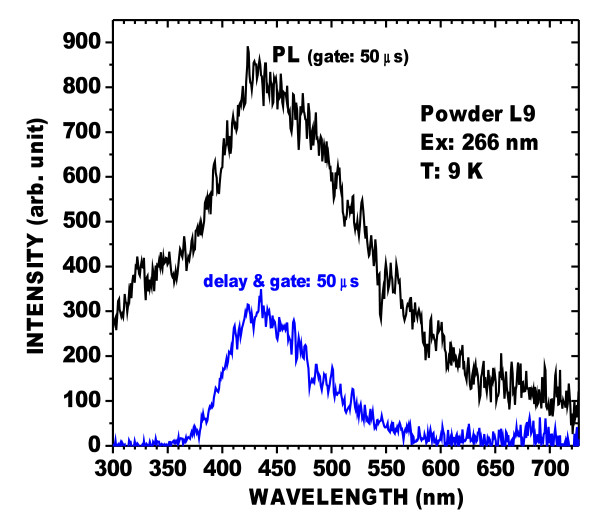
**PL spectra of powder L9 after HWA treatment at 9 K.** The gate time was 50 μs for both spectra. The strongest PL spectrum was recorded during a laser pulse (duration, 12 ps; wavelength, 266 nm) and is labeled as ‘PL’. The other spectrum was taken 50 μs after the end of the laser pulse.

**Figure 6:**
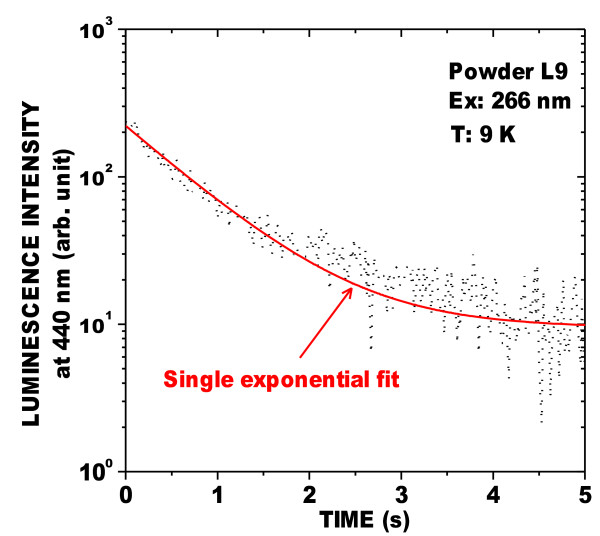
**Decay of the luminescence at 440 nm of powder L9 after HWA treatment.** At 9 K after the excitation at 266 nm was stopped. The exponential fit (solid line) gives a lifetime of about 1 s.

The blue band of powder L9 was investigated further. The PL at low temperature is very much stronger than that at room temperature (Figure 4). Moreover, the blue band included a long-lived phosphorescent component at 9 K, as shown in Figures 5 and 6. Figure 5 shows the PL, and a spectrum (long-lived component) acquired 50 μs after the excitation was stopped. Figure 6 shows that the decay of the peak of the long-lived component of the blue band, at about 440 nm, lasts several seconds. This phenomenon has been observed in oxidized PSi layers [9] for which the phosphorescence could be observed without significant changes at temperatures up to about 200 K. This confirms that the blue band observed in these powders is the same as that found in oxidized PSi layers. It is characterized by a large Stokes shift and a long-lived emission component involving triplet levels from molecular-like species.

## Conclusions

Mesoporous nanocrystalline silicon powders were fabricated using the anodization technique followed by mechanical fragmentation. HWA was used to enhance and stabilize their luminescence.

Depending on initial porosity, different color powders could be achieved: dark brown, pale brown, and off-white. The pale-brown and off-white powders exhibited mostly red and blue luminescence, respectively. The blue emission exhibits a very long lifetime (several seconds), as for previously studied lightly doped PSi layers. Such powders could have novel applications in cosmetics if the quantum efficiency can be optimized and more economic and scalable fabrication routes are developed.

## Competing interests

The authors declare that they have no competing interests.

## Authors’ contributions

BG and NK carried out the optical measurements. BG and LC carried out the high-pressure water vapor annealing. AL prepared the porous silicon powders and carried out the BET measurements. LC and NK participated in coordination. BG wrote the paper. All authors read, contributed to and approved the final manuscript.

## Authors’ information

BG is an associate professor at Nagoya University, Japan. AL is a principal scientist at pSiMedica Ltd., UK. LTC is a chief scientific officer at pSiMedica Ltd. NK is a professor at Tokyo University of Agriculture and Technology.
